# Protective effects of a lactobacilli mixture against Alzheimer’s disease-like pathology triggered by *Porphyromonas gingivalis*

**DOI:** 10.1038/s41598-024-77853-1

**Published:** 2024-11-08

**Authors:** Niloofar Kazemi, Mohammad Rabbani Khorasgani, Maryam Noorbakhshnia, Seyed Mohammad Razavi, Tahmineh Narimani, Narges Naghsh

**Affiliations:** 1 Department of Cell and Molecular Biology and Microbiology, Faculty of Biological Science and Technology, University of Isfahan, Isfahan, Iran; 2 Department of Plant and Animal Biology, Faculty of Biological Science and Technology, University of Isfahan, Isfahan, Iran; 3 Department of Oral and Maxillofacial Pathology, Isfahan University of Medical Sciences, Isfahan, Iran; 4 Department of Bacteriology and Virology, Faculty of Medicine, Isfahan University of Medical Sciences, Isfahan, Iran; 5 Department of Periodontology, Torabinejad Dental Research Center, Isfahan University of Medical Sciences, Isfahan, Iran

**Keywords:** Lactobacilli, *Porphyromonas gingivalis*, Alzheimer’s disease-like pathology, Gingival inflammation, Neuroinflammation, Rat, Microbiology, Neuroscience, Physiology

## Abstract

*Porphyromonas gingivalis* (*P. gingivalis*) is one of the pathogens involved in gingival inflammation, which may trigger neuroinflammatory diseases such as Alzheimer’s disease (AD). This study aimed to investigate the protective (preventive and treatment) effects of a lactobacilli mixture combining *Lactobacillus reuteri* PTCC1655, *Lactobacillus brevis* CD0817, *Lacticaseibacillus rhamnosus* PTCC1637, and *Lactobacillus plantarum* PTCC1058 against *P. gingivalis*-induced gingival inflammation and AD-like pathology in rats. These probiotic strains exhibited cognitive enhancement effects, but this study proposed to assess their activity in a mixture. To propose a probable mechanism for *P. gingivalis* cognitive impairments, the TEs balance were analyzed in hippocampus and cortex tissues. Animals were divided into five groups: the control, lactobacilli, *P. gingivalis*, lactobacilli + *P. gingivalis* (prevention), and *P. gingivalis* + lactobacilli group (treatment) groups. The behavioral and histopathological changes were compared among them. Finally, The Trace elements (TEs) levels in the hippocampus and cortex tissues were analyzed. The palatal tissue sections of the *P. gingivalis* infected rats showed moderate inflammation with dense infiltration of inflammatory cells, a limited area of tissue edema, and vascular congestion. Additionally, passive avoidance learning and spatial memory were impaired. Histopathological tests revealed the presence of Aβ-positive cells in the P. gingivalis group. While the Aβ-positive cells decreased in the treatment group, their formation was inhibited in the preventive group. Administration of a mixture of lactobacilli (orally) effectively mitigated the gingival inflammation, Aβ production, and improved learning and memory functions. Moreover, Zn, Cu, and Mn levels in the hippocampus were dramatically elevated by *P. gingivalis* infection, whereas lactobacilli mixture mitigated these disruptive effects. The lactobacilli mixture significantly prevented the disruptive effects of *P. gingivalis* on gingival and brain tissues in rats. Therefore, new formulated combination of lactobacilli may be a good candidate for inhibiting the *P. gingivalis* infection and its subsequent cognitive effects. The current study aimed to evaluate the effects of a lactobacilli mixture to manage the disruptive effects of *P. gingivalis* infection on memory.

## Introduction

Alzheimer’s disease (AD) is a multifactorial brain disorder that impairs memory and thinking skills and, eventually, the ability to perform basic tasks^1^. Neuroinflammation has long been thought to be the driving force behind AD, which can be triggered by infection^2,3^. Recently, the possible role of microbial pathogenesis in AD has received considerable attention. Also, the discovery of antimicrobial properties in the Amyloid beta (Aβ) peptide has supported the infectious etiology of AD and a growing interest in identifying the likely AD-related infectious agents has been developed^4^.

Periodontitis is a chronic disease that may be linked to several systemic diseases and is associated with the production of biomarkers like B-type natriuretic propeptide (NT-proBNP), α1-antitrypsin, C-reactive protein (hs-CRP), and endothelial cell-specific molecule-1 (ECM-1)^5^. In addition, biomarkers like transforming growth factor-β1 (TGF-β1) and vascular endothelial growth factor (VEGF) play a crucial role in periodontitis development^6^. Evidence shows a strong link between Alzheimer’s disease, immunological mediators, and antibodies against periodontal bacteria^7^. A 6-month study of AD patients with active periodontitis revealed a significant decline in cognition compared to AD patients without active periodontitis, raising doubts regarding the probable mechanisms behind these findings^8^. In the quest to find the oral bacterial agent affecting AD, periodontitis was evoked by *P. gingivalis* in a transgenic mouse model of AD. The study results revealed that *P. gingivalis* oral infection decreases cognitive performance, increases the deposition of AD-like plaques, and causes alveolar bone loss^9^. *P. gingivalis*, a gram-negative anaerobic bacterium, is the predominating agent in periodontitis, leading to gingival erythema, edema, and disruption of gingival tissue^7^. Infection with *P. gingivalis* may promote inflammatory mediator production and activate the complement system through systemic inflammation and elevated levels of inflammatory cytokines in the brain tissue, which can cause memory loss^10,11^. In addition, the isolation of *P. gingivalis* lipopolysaccharide (LPS) from the brains of AD patients may indicate its contribution to AD development^10^. Inhibition and eradication of *P. gingivalis*-induced periodontal infections may reduce the risk of periodontitis expression and systemic infections related to AD development.

On the other hand, treating periodontitis, especially those resulting from biofilm-forming bacteria, is difficult. Therefore, finding effective antibiotic alternatives or new preventive approaches is required^10^. The effectiveness of regular preventive strategies in inhibiting the spread of oral cavity infection is widely acknowledged in the literature^12^. Probiotics prevent infection by strengthening the epithelial barriers, depriving the pathogens of nutrients, and producing antimicrobial products such as short-chain fatty acids (SCFAs). They may also strengthen the gut-brain axis by modulating various signaling pathways, such as metabolic, oxidative, and immunologic pathways^13^. Studies revealed that *L. plantarum* MTCC1325 ameliorated cognition deficits after sixty days in D-galactose-treated albino rats^14^. Additionally, there is a link between the type of periodontitis treatment in periodontitis patients and the reduction of the production of the cardiovascular risk mediators such as C-reactive protein (CRP). The studies suggested that receiving minimally invasive non-surgical therapy (MINST) led to a greater reduction in CRP level in patients compared to quadrantwise subgingival instrumentation (Q-SI) after 1 year^15^. Moreover, a 6-month follow-up study revealed one-stage full-mouth subgingival instrumentation (FM-SI) was more effective than the Q-SI in reducing the mean probing pocket depth (PPD) and number of periodontal pathogens in periodontitis patients^16^. On the other hand, the administration of probiotics as prevention or treatment could be considered a non-surgical strategy against periodontitis, which may decrease the inflammation mediator production due to their anti-inflammatory effects^15^.

Similarly, oral administration of *Lactobacillus pentosus* var. plantarum C29 rescued memory impairment in D-galactose-treated male C57BL/6J mice and significantly decreased the inflammation markers’ expression^16^. The formulated mixture of probiotics has been shown to significantly reduce oxidative stress in the brain of AD mouse models. Administration of mixed probiotics formulation with nine live microorganisms from the Bifidobacteriaceae and Lactobacillaceae, namely SLAB51, significantly mitigated oxidative stress in AD mice through the activation of the SIRT1 pathway^17^. Formulation of probiotics with a combination of *L. plantarum* NCIMB 8826, *Lactobacillus fermentum* NCIMB 5221, and *Bifidobacterium longum* sp. infantis NCIMB 702,255 significantly ameliorates neurodegeneration by decreasing the AD development in a transgenic humanized *Drosophila melanogaster* model of AD^18^. Taken together, the in vivo studies have shown that the administration of probiotics ameliorates cognitive impairments, suppresses oxidative stress-responsive elements, and modulates the expression of regulatory genes in animal models.

Another critical aspect of cognitive impairment is the homeostatic equilibrium of brain trace elements (TEs). TEs play essential roles in biological systems due to their crucial function in various biochemical and physiological processes^19^. TEs such as copper (Cu), zinc (Zn), iron (Fe), and manganese (Mn) provide prevention against the generation of reactive oxygen species (ROS) in the brain. They function as co-factors for ROS-scavenging enzymes such as superoxide dismutase and glutathione reductase, and they modulate oxidative tissue damage in response to infection^20^. Metal ion homeostasis is essential for the brain to function properly. Alteration of homeostatic concentration of TEs is known as a risk factor for the development of AD, Parkinson’s, and Huntington’s diseases^21^. Excessive amounts of these vital elements in the brain may lead to cellular damage and various syndromes, including AD^22^. Infections, on the other hand, are considered a risk factor for TEs imbalance and the establishment of an inflammatory environment in the brain.

Despite the fact that multiple studies have documented the positive effects of probiotics on memory and gingival inflammation, there is no data regarding the benefits of probiotics on the AD-like pathology triggered by *P. gingivalis* infection^23^. The current study aimed to evaluate the effects of a lactobacilli mixture (with a combination of *Lactobacillus reuteri* PTCC1655, *Lactobacillus brevis* CD0817, *Lacticaseibacillus rhamnosus* PTCC1637, and *Lactobacillus plantarum* PTCC1058) to manage the disruptive effects of *P. gingivalis* infection on memory. In addition, we investigated the disruptive effects of *P. gingivalis* infection on the TEs homeostasis in the cortex and hippocampus. Therefore, we assessed the cognitive, histopathologic, and TE changes fallowing the *P. gingivalis* infection. The results were compared with animals administered the lactobacilli mixture to investigate the improving effects of them in both preventive and treatment groups. The current study aimed to evaluate the effects of a lactobacilli mixture to prevent and manage the disruptive effects of *P. gingivalis* infection on memory.

## Materials and methods

### Study design

**Fig. 1:**
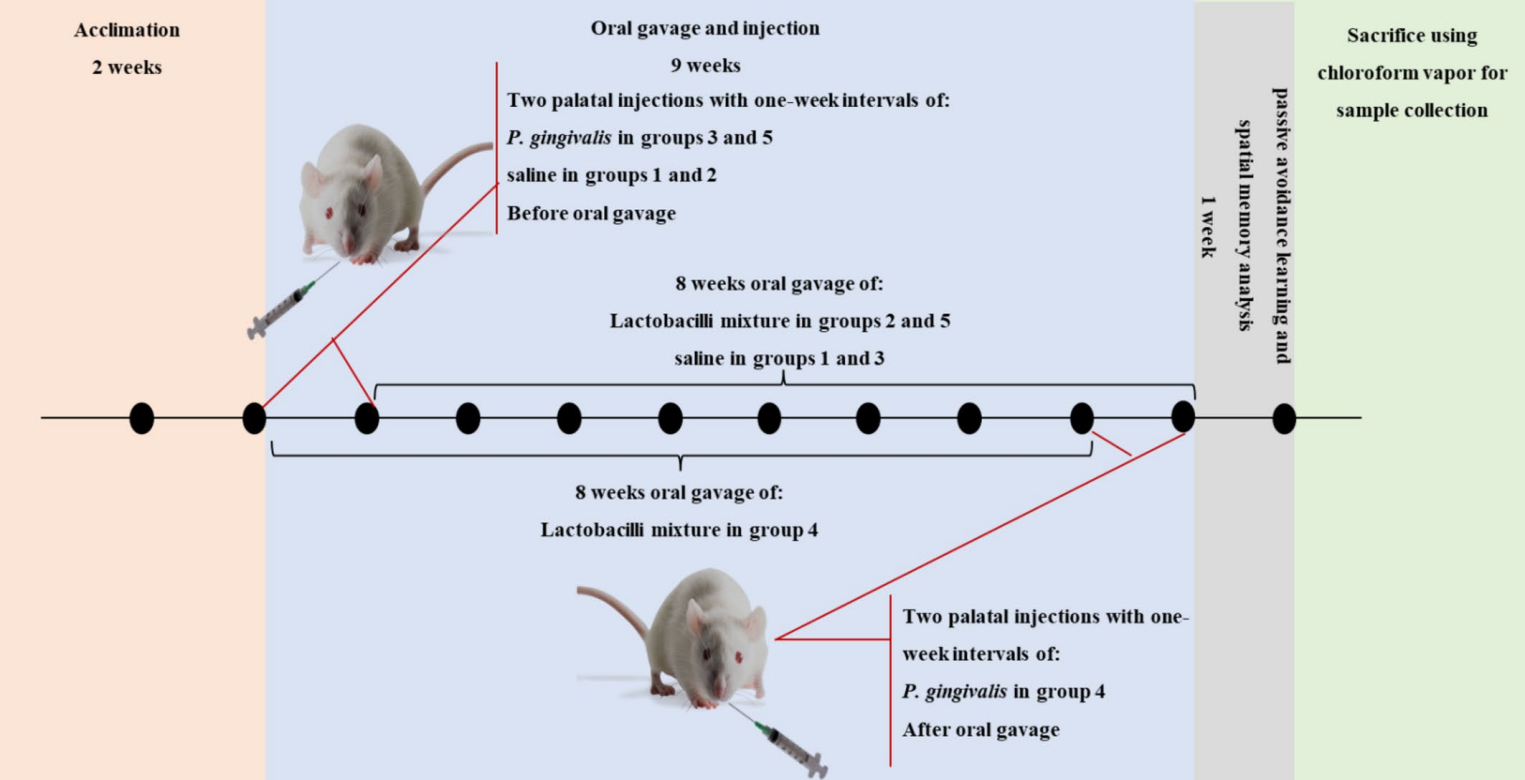
Experimental design. After 2 weeks of acclimation, rats received two palatal injections of *P. gingivalis* in groups 3 and 5 and saline in groups 1 and 2. The animals then received 8 weeks oral gavage of lactobacilli mixture in groups 2 and 5 and saline in groups 1 and 3. The rats received two palatal injections of *P. gingivalis* following the 8 weeks of oral gavage of lactobacilli in group 4.

Animals were randomly divided into five groups (Fig. 1), with eight rats per group. Groups were categorized as follows:

Group 1: The control group received two palatal injections of saline and then 8 weeks of oral gavage of saline.

Group 2: The lactobacilli group received two palatal injections of saline and then 8 weeks of oral gavage of lactobacilli.

Group 3: The *P. gingivalis* group received two palatal injections of *P. gingivalis* and then 8 weeks of oral gavage of saline.

Group 4: The lactobacilli + *P. gingivalis* group (prevention group) received two palatal injections of *P. gingivalis* following the 8 weeks of oral gavage of lactobacilli.

Group 5: The *P. gingivalis* + lactobacilli group (treatment group) received 8 weeks of oral gavage of lactobacilli following two palatal injections of *P. gingivalis*.

The animals were sacrificed using chloroform vapor for histopathological analysis.

Animals that exhibited severe weight loss, anorexia, weakness, and died due to unknown factors were excluded from the study.

## Animals

Forty-five male Wistar rats (weighing 80–100 g, 4 weeks old) were obtained from the Department of Plant and Animal Biology breeding colony, University of Isfahan. Four rats were housed in each cage with free access to food and water in a controlled condition under a 12-light/12-dark-hour cycle at 23^°^C. Animals were kept according to the guidelines for the Care and Use of Animals in Research (8th edition, National Academies Press, 2011), and all the procedures were approved by the Ethics Committee of the Faculty of Biological Science and Technology, the University of Isfahan (ethical approval ID: IR-UI-REC.1400.126).

### *P. gingivalis* preparation and administration

*P. gingivalis* ATCC32277 from − 80^°^C frozen glycerol stock was cultivated on Columbia agar supplemented with 5% sheep blood, 0.1 µg/ml vitamin K1, and 5 µg/ml hemin and incubated at 37^°^C in an anaerobic jar for 2 to 3 days. The strain was grown in an oxygen-free environment (10% CO_2_, 10% H2, and 80% N_2_) using the gas-pack and Anoxomat systems^24,25^. The bacteria were washed in sterile Phosphate-buffered saline (PBS) and prepared at a final concentration of 10^10^ CFU/ml. 100 µl of the prepared solution was injected into the palatal gingival tissues between the first and second molar, and this procedure was performed again after 7 days (each rat received two injections). In the control group, animals were injected with 100 µl of sterile saline in the palatal gingiva. During the procedure, rats were anesthetized with an intraperitoneal injection of ketamine (80 mg/kg) and xylazine (20 mg/kg). After 45 days, behavioral tests were done to evaluate passive avoidance learning and spatial memory abilities^26^.

## Lactobacilli preparation and administration

The bacterial strains from lactobacilli (*Lactobacillus reuteri* PTCC1655, *Lactobacillus brevis* CD0817, *Lacticaseibacillus rhamnosus* PTCC1637, and *Lactobacillus plantarum* PTCC1058) were cultured in MRS broth and then incubated at 37^°^C in an anaerobic jar for 24 h. The bacterial strains were then streaked on MRS agar under the same conditions^27^. Daily fresh lactobacilli cultures were prepared and harvested in sterile saline by centrifugation, and the supernatant was removed. The procedure was repeated to remove the debris and the cells suspended in sterile saline. The optical density of the lactobacilli mixture was read at 600 nm, and oral gavage was done (10^9^ CFU/ml per day) for the groups for eight weeks. In the control group, animals received saline^23,27^.

## Behavioral tests

### Morris water maze test

The Morris water maze (MWM) test evaluated spatial learning and memory abilities. In this test, animals search for a hidden, fixed platform 2 cm below the water’s surface in a certain quadrant of a black pool. The black circular pool (150 cm) was filled with 25 °C water and divided into four quadrants. Spatially observable clues were also installed around the pool so that animals could use them to recognize the path of the target platform. Rats were trained for 3 consecutive days with 4 trial sessions per day as the acquisition phase. Each experiment started with releasing an animal facing the pool’s wall in one of the four quadrants and allowing it to swim for 60 s to locate the invisible platform. The probe test was assessed after the platform removal on the fourth day. The escape latency and distance traveled to reach the platform are measured to evaluate the animal’s learning ability during training days, and the animals then must recall the target area in the probe test^28^.

## Passive avoidance test

The passive avoidance apparatus comprises two illuminated and dark enclosures joined by a guillotine door. Each rat was placed in the illuminated enclosure facing away from the guillotine for 15 s, and then the door was opened. As the rat entered the dark box, the door was closed, and the rat was permitted to stay there for 30 s. The door was opened, and the rat returned to the home cage. The trial test was performed after 30 min immediately following the animal’s entrance to the dark enclosure by receiving a foot shock (1/1 mA, 50 Hz, 0/8 s), and doors were then opened to return to the illuminated enclosure after 30 s. The same procedure was repeated until the rat stayed in the light part for 120 s. The retention test was done a day after the acquisition test with the same method, but the doors were kept up without exerting foot shock. Step-through latency (STL) and the amount of time spent in the dark component (TDC) were recorded for 10 min^29^.

### Brain tissue preparation

Finally, the animals were sacrificed, and their brains were immediately collected. The hippocampus and cortex tissues were then separated and kept in a 10% formalin buffer for further analysis. A tissue processor automatically dehydrated the tissue samples, fixed them, and infiltrated them in paraffin (BC-6800 Plus). The serial sections were cut at 6 μm using a microtome (Accu-Cut SRM). The slides were stained with Congo red to observe the amyloid plaques and another histopathological alteration^30,31^.

## Gingival tissue preparation

The palatal gingival tissues were separated and stored in a 10% formalin buffer. Moreover, the slide sections were prepared using the same techniques as the brain tissue. All the samples were counterstained by hematoxylin and eosin (H & E)^30,32^. Referring to Cox and Rabin’s criteria the inflammatory tissue responses score was evaluated^33,34^.

## Trace elements evaluation

The TEs levels (Fe, Cu, Mn, and Zn) were determined using atomic absorption (Shimadzu AA- 670, Kyoto, Japan. The UV-visible detector was the following: Fe: 248.3 nm, Cu: 324.8 nm, Mn: 279.6 nm, and Zn: 213.9 nm. To evaluate the TE levels in the brain tissues, they were dried at 60 °C for 24 h, dissolved in a mixture of perchloric and nitric acid (3:7 ratios, respectively), and then placed in a 55 °C water bath^35^.

### Statistical analysis

Results were expressed as mean ± SEM. Data were analyzed with GraphPad Prism version 8 for Windows through one-way and two-way ANOVA (repeated measures ANOVA) followed by post-hoc Tukey’s multiple comparisons tests. If P values were below 0.05, then the data was considered significant.

## Results

### Effect of lactobacilli and *P. gingivalis* on spatial memory

**Fig. 2:**
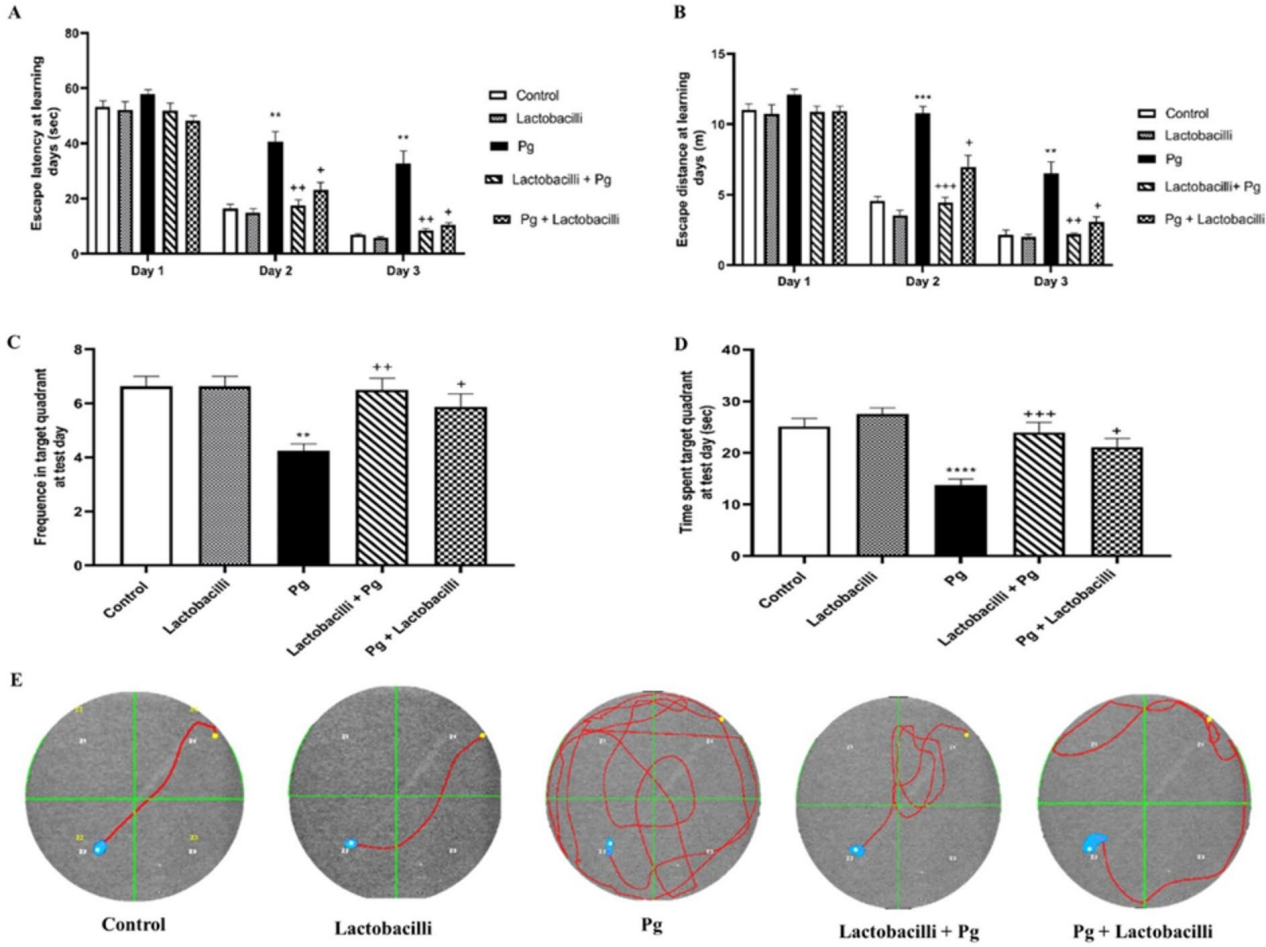
Comparison of behavioral changes among studied groups in MWM test. (A) Escape latency. (B) The distance traveled by rats to reach the platform during learning days. (C) The number of times crossing the target area in the probe trial. (D) The time spent in the target quadrant in the probe test. (E) The representative trajectory for each group in the probe test. Data were expressed as means ± SEM, (*n* = 8), Pg = *Porphyromonas gingivalis*, lactobacilli + Pg = prevention group, Pg + lactobacilli = treatment group. *** denotes P<0.001 and ** denotes P<0.01 when compared to control group. +++ denotes P<0.001, ++ denotes P<0.01 and + denotes P<0.05 when compared to P. gingivalis-infected group.

The MWM test was applied to evaluate spatial learning and memory. Two-way repeated-measures ANOVA revealed a considerable increase in both escape latency and distance traveled to reach the platform during days 2 (P<0.01 and P<0.001 respectively) and 3 (P<0.01 and P<0.01 respectively) of training in *P. gingivalis* group compared to the control (Fig. 2A and B respectively). Also, one-way ANOVA followed by post hoc analysis showed that injection of *P. gingivalis* caused a significant reduction in the crossing number of the target area (*P* < 0.01) and time spent in the target quadrant (*P* < 0.0001) concerning the control group in probe test (Fig. 2C and D respectively). The results of training tests (learning evaluation) and probe trials (memory evaluation) strongly supported the memory impairment development in *P. gingivalis*-infected rats.

Compared to the *P. gingivalis* group, lactobacilli administration in lactobacilli + *P. gingivalis* (preventive group) and *P. gingivalis* + lactobacilli (Treatment group) groups significantly improved escape latency (P<0.01 and P<0.05 respectively) and distance traveled to reach the platform during days 2 (P<0.001 and P<0.05 respectively) and 3 (P<0.01 and P<0.05 respectively) (Fig. 2A and B).

According to probe test results, lactobacilli + *P. gingivalis* and *P. gingivalis* + lactobacilli groups significantly improved performance in remembering the platform’s location. One-way ANOVA revealed a significant increase in the number of crossings of the target area and time spent in the target quadrant in lactobacilli + *P. gingivalis* (*P* < 0.05) and *P. gingivalis* + lactobacilli groups (P<0.01and P<0.001, respectively) compared to the *P. gingivalis* group (Fig. 2C and D respectively). Moreover, the probe test day representative trajectory of studied groups indicated that lactobacilli + *P. gingivalis* and *P. gingivalis* + lactobacilli groups explored fewer non-target quadrants than the *P. gingivalis* group (Fig. 2E). There was no significant difference between the control and lactobacilli-administrated groups in all examined MWM parameters.

In general, the results revealed that *P. gingivalis* significantly impaired spatial learning and memory in the MWM task, while lactobacilli supplementation rescued *P. gingivalis*-induced MWM task impairments in both lactobacilli + *P. gingivalis* and *P. gingivalis* + lactobacilli groups. Notably, the improvement effects of lactobacilli supplementation were more prominent in the lactobacilli + *P. gingivalis* group.

### Effect of lactobacilli and *P. gingivalis* on passive avoidance learning

**Fig. 3:**
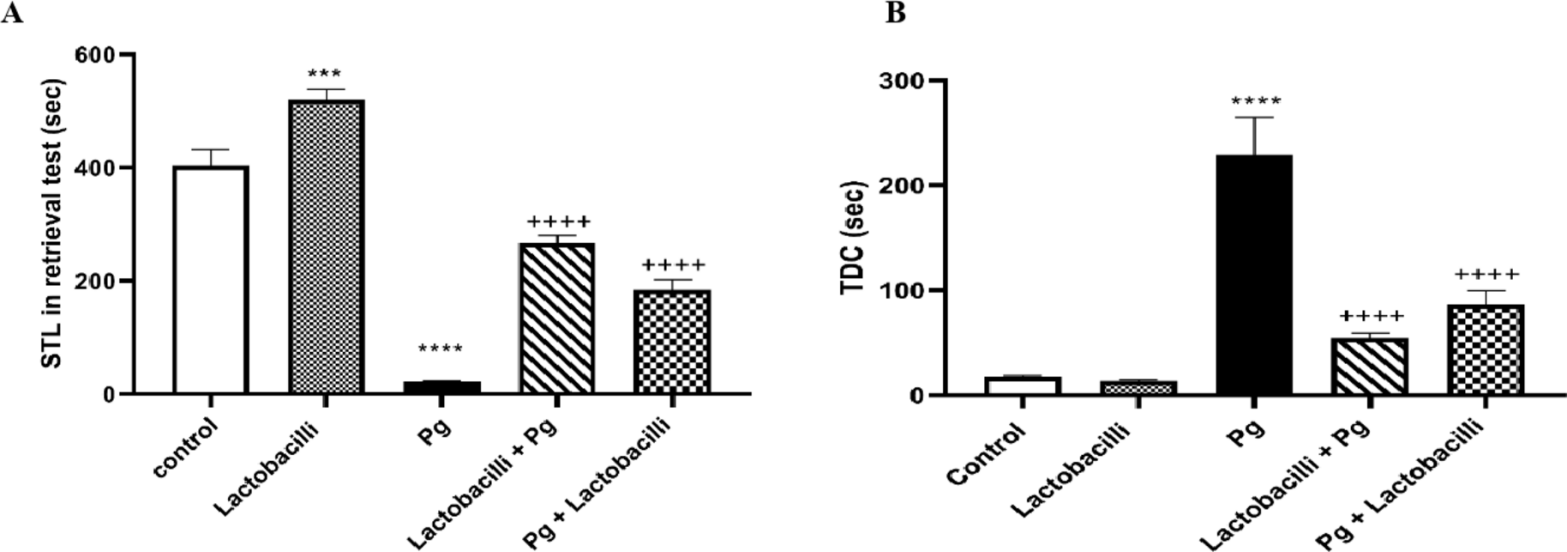
Effects of P. gingivalis infection and lactobacilli-administration on passive avoidance learning and memory performance. (A) step-through latency (STL) in studied groups, and (B) time spent in the dark compartment (TDC) applied on the following day of the acquisition trial. Data were expressed as means ± SEM, (*n* = 8), Pg = *Porphyromonas gingivalis*, lactobacilli + Pg = prevention group, Pg + lactobacilli = treatment group. **** denotes P<0.0001 and *** denotes P<0.001 when compared to control group. ++++ denotes P<0.0001when compared to *P. gingivalis*-administrated group.

One-way ANOVA followed by post-test analysis revealed significant differences in STL between the control group and the lactobacilli group (P<0.001) (Fig. 3A), demonstrating the beneficial effect of lactobacilli on memory function. The STL test revealed a reduction (P<0.0001) in the *P. gingivalis* group compared to the control group, indicating the impairment of STL following *P. gingivalis* induction) (Fig. 3A). The TDC increased in the *P. gingivalis* group compared to the control (P<0.0001) (Fig. 3B), indicating the impairment effect of *P. gingivalis* on memory performance. Both lactobacilli + *P. gingivalis* and *P. gingivalis* + lactobacilli groups showed improved learning and memory evaluation. Moreover, STL results revealed that lactobacilli mixture administration in both lactobacilli + *P. gingivalis* and *P. gingivalis* + lactobacilli groups (*P* < 0.0001 in both) significantly improved this memory parameter compared to the *P. gingivalis* group (Fig. 3A). Also, the *P. gingivalis* group showed a significant increase in the time spent in the dark compartment (P<0.001) compared to lactobacilli + *P. gingivalis* and *P. gingivalis* + lactobacilli groups (Fig. 3B).

In general, results of passive avoidance learning and memory indicated that *P. gingivalis* significantly impaired the STL and TDC function, while lactobacilli administration significantly rescued both STL and TDC.

### Histopathological changes in brain tissue

**Fig. 4:**
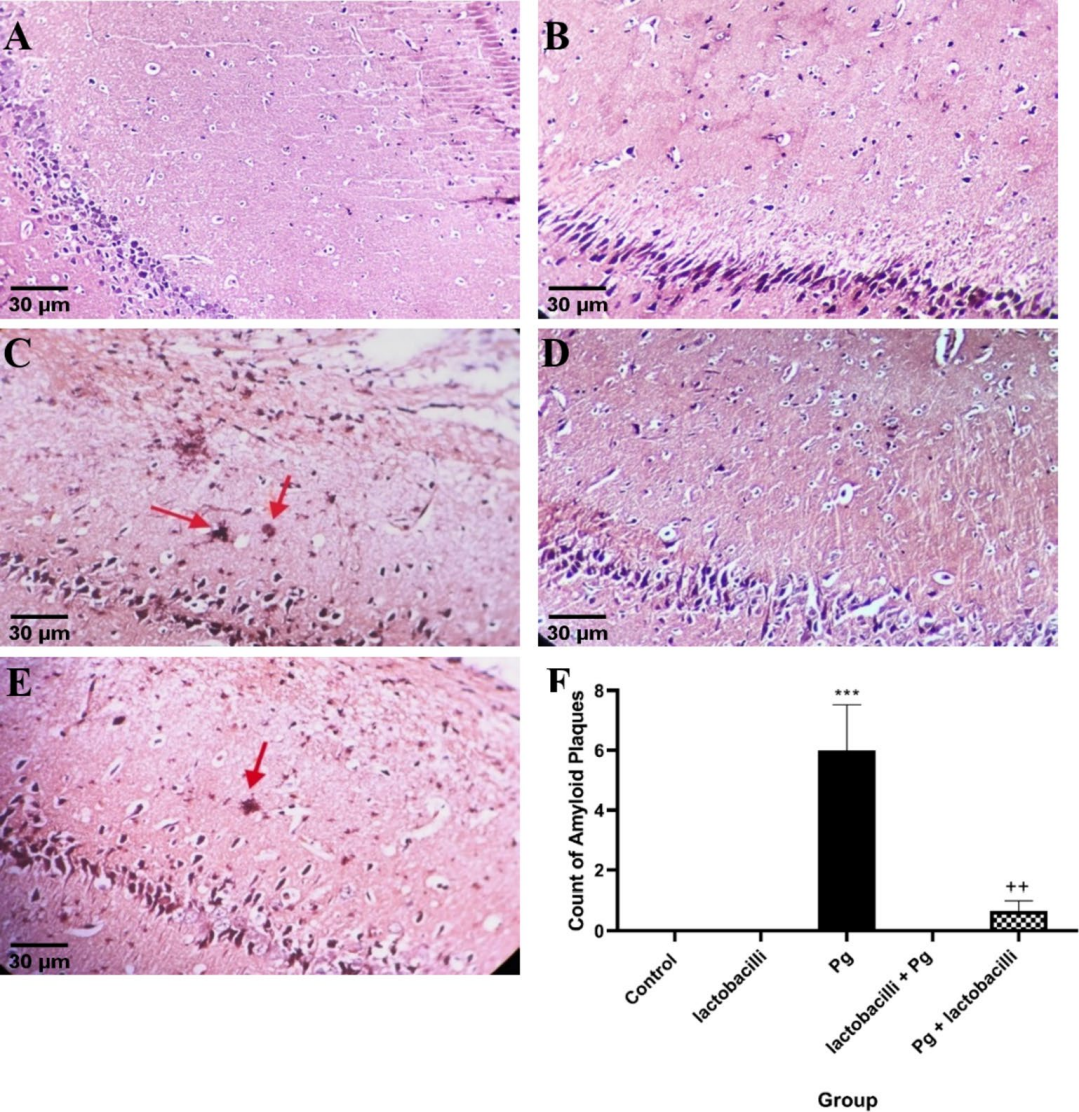
Congo red-stained hippocampus sections of all studied groups. The red arrows indicated the Aβ plaques. The micrograph represented (A) control, (B) lactobacilli, (C) P. gingivalis, (D) lactobacilli + P. gingivalis; and (E) P. gingivalis + lactobacilli and (F) Count of amyloid plaques. Data were expressed as means ± SEM, (*n* = 3), Pg = *Porphyromonas gingivalis*, lactobacilli + Pg = prevention group, Pg + lactobacilli = treatment group. *** denotes P<0.001 when compared to the control group. ++ indicates P<0.01when compared to *P. gingivalis*-administrated group. Representative images of three sections per hippocampus and three brains per group were observed with 20x objectives.

Figure 4 illustrates Congo red-stained brain sections in all studied groups to assess the number of Aβ plaques in the hippocampus. Normal histopathological structure without any alteration was observed in control (Fig. 4A) and lactobacilli groups (Fig. 4B). In contrast, histopathological changes were noticed in the hippocampal sections of the *P. gingivalis* group. The Aβ-positive cells were only observed in the *P. gingivalis* and *P. gingivalis* + lactobacilli groups (Fig. 4C and E, respectively). A hippocampal-widespread distribution of Aβ plaques in the *P. gingivalis* group following *P. gingivalis* injection indicated the association of this pathogen with the presence of AD-like pathology (Fig. 4C). Fewer Aβ-positive cells were detected in the hippocampal sections of the *P. gingivalis* + lactobacilli group with respect to the *P. gingivalis group*, indicating lactobacilli supplementation decreased the *P. gingivalis*-induced hippocampal damage (Fig. 4E). On the other hand, no Aβ-positive cells were observed in the lactobacilli + *P. gingivalis* group (Fig. 4D).

### Pathologic analysis of periodontal tissue

**Fig. 5:**
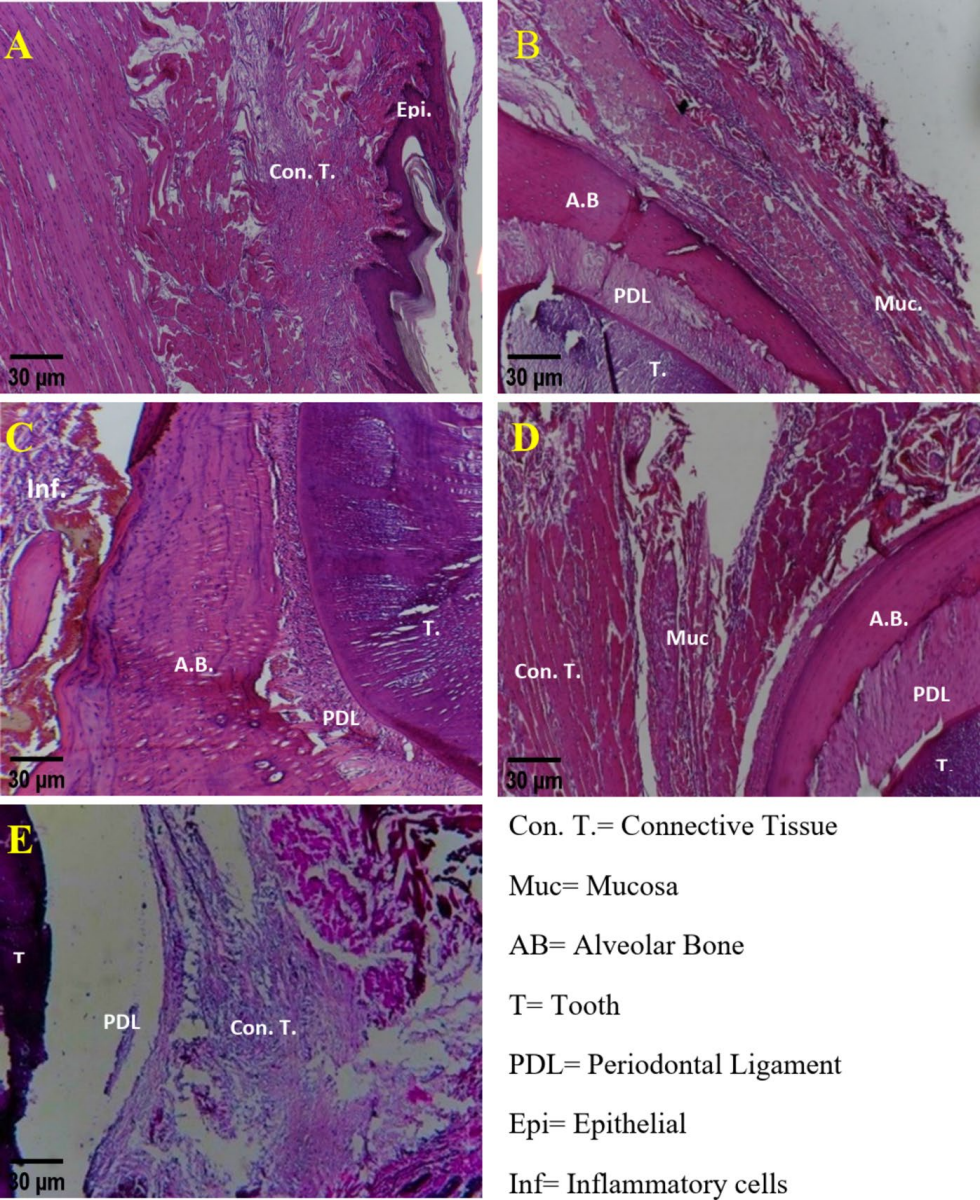
H&E-stained palatal gingival tissue sections in all studied groups. The micrograph represented: (A) control; (B) lactobacilli; (C) P. gingivalis; (D) lactobacilli + P. gingivalis; and (E) P. gingivalis + lactobacilli. Histopathological analysis showed moderate inflammation in the palatal gingival tissue of P. gingivalis-infected rats and mild inflammation in *P. gingivalis* + lactobacilli group. In contrast, no histopathological alteration was observed in the control, lactobacilli, and lactobacilli + *P. gingivalis* groups. Representative images of three sections per palatal gingival tissue and three gingival tissues per group were observed with 20x objectives.

To determine the pathologic effects of *P. gingivalis infection*, an H&E-stained periodontal section from the palatal gingival tissue was prepared and examined. The inflammatory responses were determined by inflammatory cells, tissue deposition or fibrosis, edematous areas, and vascular alterations in the palatal gingival tissue. The palatal tissue sections of the *P. gingivalis* group showed moderate inflammation with dense infiltration of inflammatory cells, a limited area of tissue edema, and vascular congestion (Fig. 5C). Moreover, to determine the ameliorative and protective effects of formulated lactobacilli against the *P. gingivalis*-induced infection, the inflammation parameters were evaluated in lactobacilli + *P. gingivalis* and *P. gingivalis* + lactobacilli groups. Mild inflammation was detected in the *P. gingivalis* + lactobacilli group due to a mild infiltration of inflammatory cells, wavy collagen fiber deposits, and fibrosis (Fig. 5E). Interestingly, no histopathological alteration was confirmed in the lactobacilli + *P. gingivalis* group (Fig. 5D). Also, pathological analysis revealed no significant changes in the gingival tissue of the control and lactobacilli groups (Fig. 5A and B, respectively).

### Effect of lactobacilli and *P. gingivalis* on trace elements

#### Hippocampus tissue

**Fig. 6:**
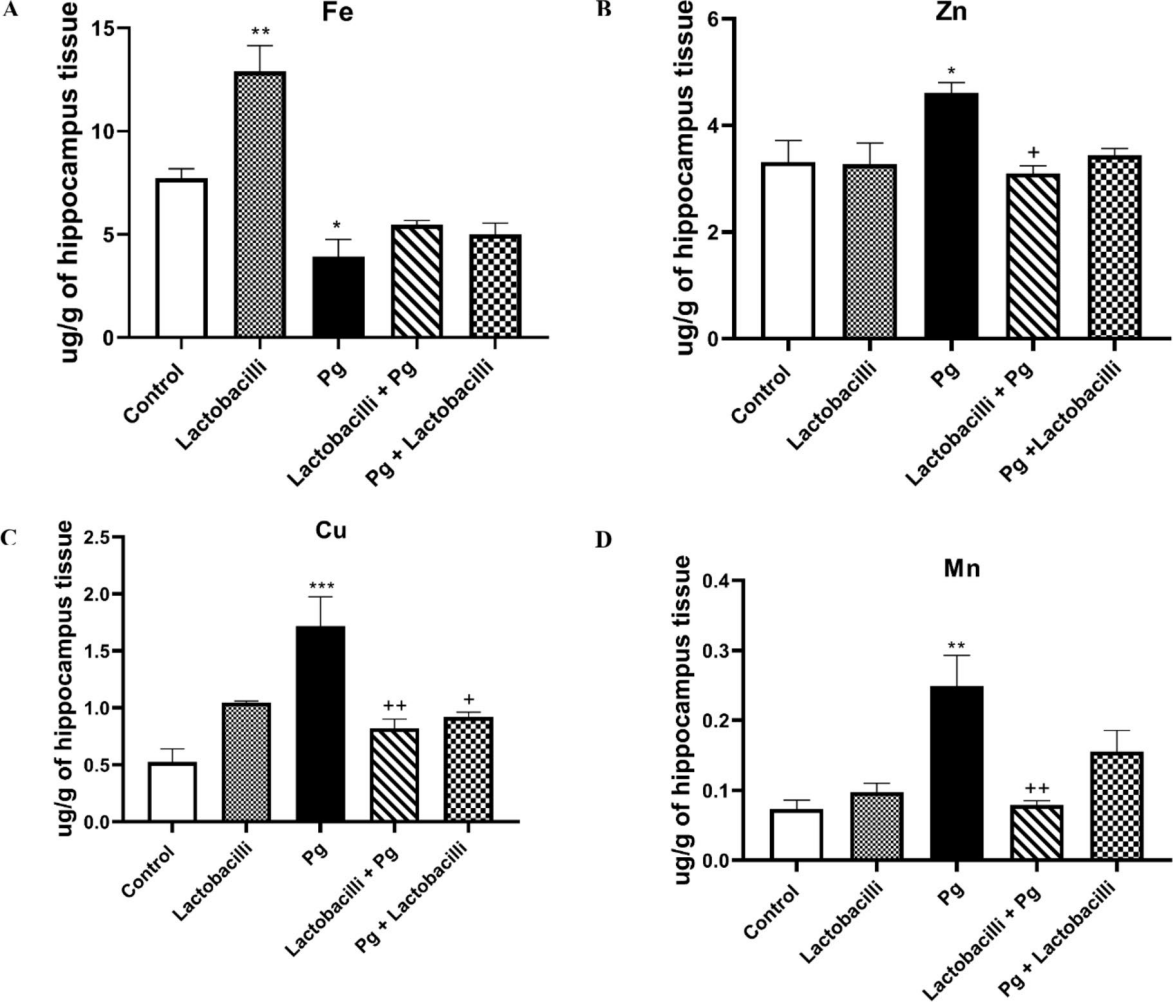
Comparison of hippocampus TEs concentrations. (A) Fe, (B) Zn, (C) Cu, and (D) Mn hippocampus levels among studied groups using atomic absorption spectrophotometry. Data showed that infection with P. gingivalis significantly altered the hippocampus trace element homeostasis, and lactobacilli supplementation modulates these changes. Data were expressed as means ± SEM, *n* = 8, Pg = *Porphyromonas gingivalis*, lactobacilli + Pg = prevention group, Pg + lactobacilli = treatment group. *** denotes P<0.001, ** denotes P<0.01, and * denotes P<0.05 when compared to control group. ++ denotes P<0.01, and + denotes P<0.05 when compared to P. gingivalis-administrated group.

Analysis of brain Fe, Cu, Zn, and Mn levels in all studied groups are depicted in Fig. 6. The obtained data showed that *P. gingivalis*-induced infection considerably altered the homeostatic equilibrium of hippocampus TEs, which may establish the development of various neuropathologic diseases such as AD. Surprisingly, lactobacilli supplementation mitigated hippocampus TEs alterations following *P. gingivalis* induction.

Statistically significant increases in the homeostatic concentrations of Zn (P<0.05), Cu (P<0.001), and Mn (P<0.01) levels were observed in the *P. gingivalis* group compared to the control group (Fig. 6B, C, and D respectively). In contrast, Fe concentration in the *P. gingivalis* group showed a significant reduction compared to the control group (P<0.05) (Fig. 6A). Except for a significant increase (P<0.01) of Fe levels in the lactobacilli group, no significant changes were noticed between other studied TEs levels compared to the control group.

Lactobacilli supplementation significantly improved the status of TEs in the hippocampus of lactobacilli + *P. gingivalis* and *P. gingivalis* + lactobacilli groups compared to the *P. gingivalis* group. Even though Fe concentration in the *P. gingivalis* group showed a significant reduction compared to the control group, there was no significant difference in both the lactobacilli + *P. gingivalis* and *P. gingivalis* + lactobacilli groups compared to *P. gingivalis* group (Fig. 6A). Infection with *P. gingivalis* significantly increased hippocampal levels of Zn, Cu, and Mn, while lactobacilli supplementation significantly rescued *P. gingivalis*-induced alteration in lactobacilli + *P. gingivalis* group (P<0.05, P<0.01 and P<0.01respectively) (Fig. 6B, C, and D respectively). Also, the lactobacilli supplement significantly moderated the increase of Cu in the *P. gingivalis* + lactobacilli group compared to the *P. gingivalis* group (P<0.05) (Fig. 6C). However, there was no significant decrease in other TES (Zn and Mn) in the *P. gingivalis* + lactobacilli group compared to the *P. gingivalis* group (Fig. 6B and D, respectively).

### Cortex tissue

**Fig. 7:**
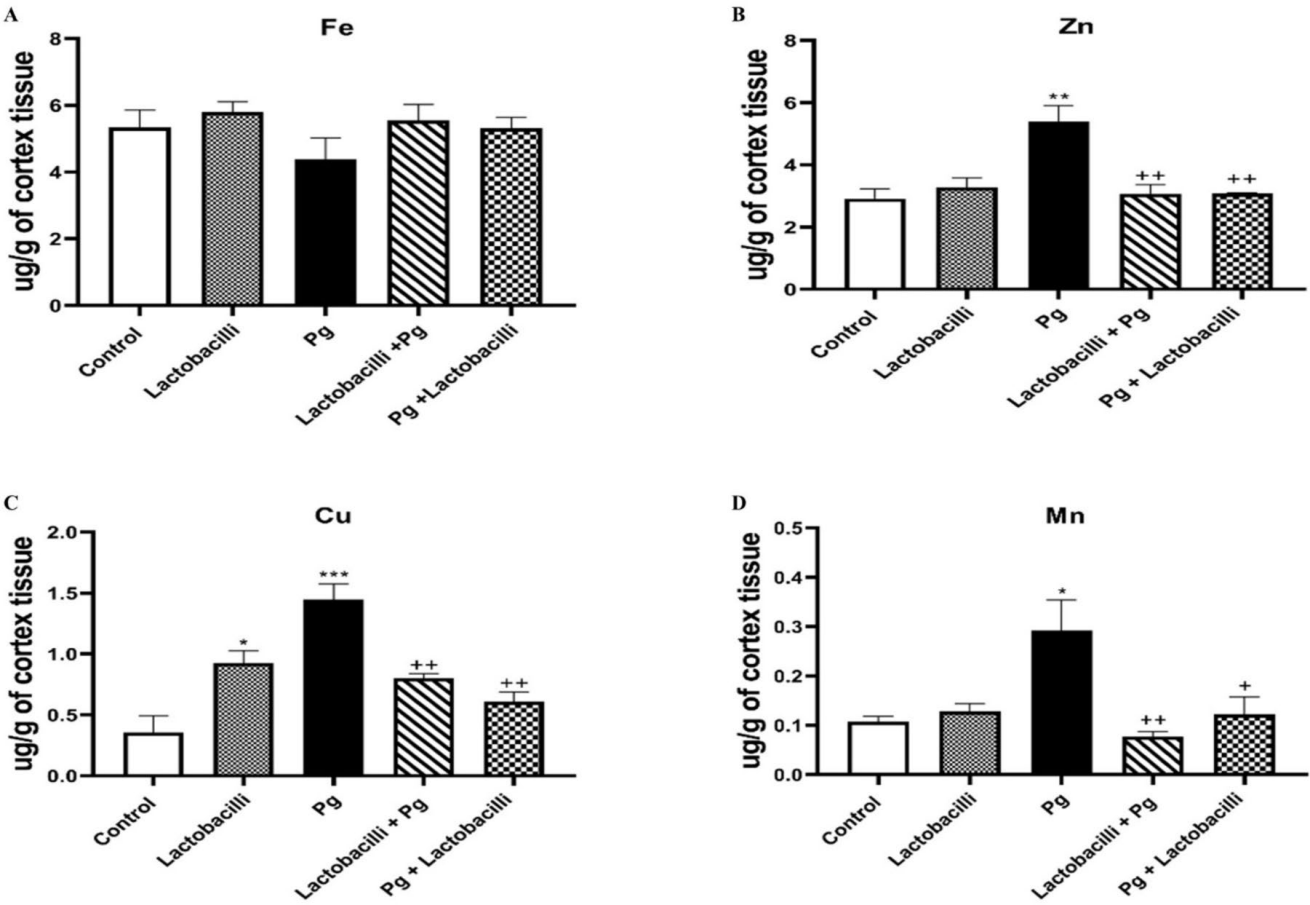
Comparison of cortex TEs concentrations: (A) Fe, (B) Zn, (C) Cu, and (D) Mn cortex levels among studied groups using atomic absorption spectrophotometry. Data revealed that P. gingivalis-induced infection noticeably altered the homeostatic concentration of TEs in the cortex. The lactobacilli supplementation significantly rescued cortex TEs alteration following P. gingivalis induction. Data were expressed as means ± SEM, *n* = 8, Pg = *Porphyromonas gingivalis*, lactobacilli + Pg = prevention group, Pg + lactobacilli = treatment group. *** denotes P<0.001, ** denotes P<0.01, and * denotes P<0.05 when compared to control group. ++ denotes P<0.01, and + denotes P<0.05 when compared to the *P. gingivalis*-administrated group.

Data obtained from Fe, Zn, Cu, and Mn levels in all studied groups are shown in Fig. 7. Statistically significant higher levels of Zn (P<0.01), Cu (P<0.001), and Mn (P<0.05) in the *P. gingivalis* group compared to the control group were noticed (Fig. 7B, C, and D, respectively). No significant difference was observed in the amount of Fe between the studied groups (Fig. 7A). As shown in Fig. 7, lactobacilli administration remarkably improved and normalized levels of Zn, Cu, and Mn following *P. gingivalis* infection in both lactobacilli + *P. gingivalis* and *P. gingivalis* + lactobacilli groups. A significant increase in Cu levels in the lactobacilli group compared to the control group (P<0.05) was noticed (Fig. 7C). Analysis of the cortex TE levels revealed that *P. gingivalis* infection considerably altered homeostatic equilibrium in some of the TEs in the cortex, similar to those in the hippocampus, which may establish the development of AD. Interestingly, lactobacilli supplementation improved and normalized the *P. gingivalis*-induced alteration in the concentration of cortex TEs.

## Discussion

Periodontitis is a major oral health problem that eventually leads to tooth loss, which can adversely affect daily activities such as eating and speaking. Not being treated early can diminish patients’ health-related quality of life (QOL)^36,37^. Periodontitis treatment decreases symptoms and disability and improves quality of life and improves oral health-related quality of life (OHRQoL)^36–38^. Therefore, there is an urgent need to detect and inhibit its causative agents. Some bacterial genera are among the most common pathogens linked to periodontitis development. Despite the fact that periodontitis is a polymicrobial infection, different studies investigated the role of specific microorganisms that contribute to the disease process^39^.

A growing body of research suggests a relationship between *P. gingivalis* infection, the most common cause of periodontitis, and accelerated cognition deficits in AD^40^. The presence of *P. gingivalis* DNA and gingival antigens in the brains of AD patients strongly supported this hypothesis^10^. Also, elevated levels of serum antibodies against periodontal bacteria confirmed that patients with periodontitis are at a higher risk of AD development^41,42^. In addition, inflammation acts as a link between periodontitis and AD. In fact, periodontitis produces pro-inflammatory factors for the systemic circulation, and the systemic inflammation promotes AD^43,44^. In addition, it has been suggested that *P. gingivalis* directly enters the brain through the blood of peripheral nerves and causes infection of the of the CNS. This infection is associated with the production of cytokines like the IL family, TNF-α, transforming growth factor-β, and chemokines, which trigger neurodegenerative disease^44^. Moreover, periodontitis conditions generate oxidative stress through an imbalance between oxidants and antioxidants, which plays an important role in the development of systemic diseases^45^. Since oxidative stress is a key player in the progression of AD disease, it seems that *P. gingivalis* infection can be linked to AD through this putative mechanism^46^. In line with previous studies, our results revealed the *P. gingivalis* infection leads to gingival inflammation, memory impairment, and AD-like pathology development in the brains of rats. The infected rats developed Aβ cells in their brain, and passive avoidance learning and spatial memory were impaired.

Previous studies have shown that probiotics have antimicrobial, modulatory, and beneficial effects against *P. gingivalis* and inflammatory diseases^47,48^. Probiotic strains from the *Bifidobacterium* and *Lactobacillus* genera have been demonstrated to block the adhesion and invasion of *P. gingivalis* to gingival epithelial cells^49^. In the current study, some protective and modulatory roles of a newly formulated lactobacilli with a combination of *Lactobacillus reuteri* PTCC1655, *Lactobacillus brevis* CD0817, *Lacticaseibacillus rhamnosus* PTCC1637, and *Lactobacillus plantarum PTCC1058* against *P. gingivalis-*related damages.

Firstly, periodontal *P. gingivalis* injections have been performed to induce gingival inflammation and, subsequently, AD-like pathology in rats. In the present study, the non-encapsulated *P. gingivalis* (ATCC32277) was used to induce gingival inflammation, and it was confirmed by pathologic findings. It is necessary to mention that in some studies, it has been hypothesized that non-encapsulated strains don’t induce inflammatory or neuroinflammatory responses^26,50^. However, Han et al. used *P. gingivalis* ATCC32277 to induce periodontal bone resorption and showed that serum IgG and salivary IgA antibodies and T cell proliferation increased in response to *P. gingivalis* infection^51^. In addition, Franka et al. suggested that the antigens of *P. gingivalis* ATCC32277 induced a humoral immune response^52^. For this reason, further studies are necessary to clarify this incompatibility.

Then, behavioral changes compared to different groups’ learning and memory abilities have been assessed. The present study revealed a significant impairment of spatial and non-spatial learning and memory function due to *P. gingivalis* palatal injection. Similarly, *P. gingivalis*-induced learning and memory impairment have been demonstrated in some studies using the Morris water maze test by increasing scape latency and decreasing the number of crossings of the targeted area^7^. Another study reported that *P. gingivalis* LPS-exposed female rats led to learning and memory impairments in the Morris water maze and shuttle box tests in both male and female offspring^53^. Also, a previous study reported learning and memory impairment following *P. gingivalis* LPS administration by neuroinflammation induction^54^. Periodontitis-induced cognitive impairments in a mouse model of AD were associated with higher levels of TNF-α and IL-1b in the brain^9^. These studies suggested neuroinflammation by releasing the pro-inflammatory cytokines TNF-α, IL-6, and IL-1β in the brain tissues as a mediate mechanism of *P. gingivalis* deleterious effects on learning and memory function. Moreover, changes in astrocytic morphology, increased Aβ1–42 levels, and Tau hyperphosphorylation in the hippocampus of young rats have been reported in the AD-like pathology triggered by *P. gingivalis* following an experimental infection period^26^. Oral chronic *P. gingivalis* application (22 weeks) in C57BL/6 WT mice has been reported to develop neuropathologic conditions associated with neuroinflammation, neurodegeneration, and extracellular Aβ1–42 production in the hippocampus^55^. It has been reported that production of Aβ40 and Aβ1–42 in neural cell cultures strongly enhanced inflammatory cytokine production (TNF-α and IL-1β) in a culture of microglial cells primed with Aβ following *P. gingivalis*-induced periodontitis^9^.

To further validate the behavioral change results, the Congo red-stained brain sections have been prepared to evaluate the number of Aβ-positive cells in the hippocampus of rats due to gingival inflammation. The histopathological analyses demonstrated *P. gingivalis*-induced Aβ plaque development and deposition in the hippocampus, which may be interpreted as evidence of neurobehavioral experiment deficits. It should be mentioned that amyloid accumulation may be found in normal people; however, this phenomenon not being observed in mouse models^56,57^. But Kobro-Flatmoen et al. found intracellular Aβ42 in neurons throughout the brain of healthy Wistar rats of all ages^58^. However, no Aβ-positive cells were observed in the control group of the current study. Therefore, measuring biomarkers like beta-amyloid 42, tau, and phospho-tau in cerebrospinal fluid (CSF) remains necessary to confirm the presence of AD^59^.

*P. gingivalis* locally colonizes periodontal tissue and provides a replicative niche, ultimately disrupting the teeth support system^60^. The inflammation-promoting effect of *P. gingivalis*-induced periodontitis has been demonstrated in mouse models by detecting higher serum levels of pro-inflammatory cytokines^60,61^. In CIA mice, the *P. gingivalis*-induced periodontitis resulted in the up-regulation of CD19 + B cells and Th17 but the down-regulation of IL-10-producing regulatory B cells (B10)^62^. It is concluded that *P. gingivalis* infection triggers inflammatory cytokine secretion, which increases tissue destruction. In the current study, the H&E-stained gingival tissue sections showed a moderate inflammation of the gingival due to *P. gingivalis* infection. While the preventive administration of the formulated lactobacilli completely prevented inflammation and histopathological alteration of gingival tissue in rats, treatment lactobacilli consumption also indicated anti-inflammatory properties. It decreased moderate inflammation to mild in the *P. gingivalis* group. Therefore, in parallel with previous studies, our results revealed the anti-inflammatory effects of lactobacilli against *P. gingivalis*^63^.

Probiotics prescription is well recognized as one of the most effective prophylactic strategies against cognitive decline in AD. Recent clinical trials and numerous in vivo studies have demonstrated the usefulness of specific bacterial strains in improving memory and reducing the progression of AD^3,64^. A study evaluated the effects of probiotic supplementation on behavioral and electrophysiological parameters of learning and memory in Aβ -induced (ICV injection of Aβ1–42 rat model of AD^65^. They reported that administration of probiotics (*Lactobacillus and Bifidobacterium* strains, by gavage) for 4 weeks before and 2 weeks after ICV injection significantly improved learning and long-term potentiation in AD rats. In an in vivo mouse model of aging (induced with D-galactose), long-term (10 weeks) application of a probiotic mixture containing *Lactobacillus paracasei* ssp. paracasei BCRC 12188, *L. plantarum* BCRC 12251, and *Streptococcus thermophilus* BCRC13869 significantly reduced the level of injury in the hippocampus, rescued learning and memory deficits, ameliorated oxidative stress, and promoted antioxidant activity in the aging mice model^66^. On the other hand, there is a positive link between non-surgical treatment and improved OHRQoL in periodontal patients^67^. Clinical parameters and Oral Health Impact Profile (OHIP)-14 scores revealed that the non-surgical treatment can significantly improve the OHRQoL in participants with periodontitis S2 and S3 after 3 months^68^. Additionally, this method led to a greater reduction in cardiovascular risk mediators’ production in patients with periodontitis^15^. The beneficial role of probiotics in the treatment of periodontal diseases could be considered an effective non-surgical therapy that doesn’t induce any pain in the patients.

Additionally, a different study found that 60 days of *L. plantarum* MTCC1325 treatment in a rat model of AD (induced by D-galactose) significantly improved spatial learning and memory in the Morris water maze task as well as gross behavioral activity, and they hypothesized that *L. plantarum* MTCC1325 has anti-Alzheimer properties against D-galactose-induced AD. It has been proposed that restoring the decreased acetylcholine level induced by D-galactose is a probiotic-mediated strategy for enhancing memory performance. Meanwhile, their histological results showed that *L. plantarum* treatment had a protective effect against Aβ proteins accumulated in the cortex and hippocampus tissue, which was supported by visibly healthy neurons with prominent nuclei^14^. Moreover, a study evaluated the effects of probiotic (*Enterococcus faecium*), prebiotic (agave inulin), or symbiotic (*E. faecium* + inulin) administration for 5 weeks on memory function and cytokine profile in a model of middle-aged rats. The results showed that the symbiotic group performed significantly better on the Morris water maze task, with reduced levels of pro-inflammatory cytokines (IL-1b and TNF-a) and higher levels of BDNF in the hippocampal tissue^69^. The current study showed that the formulated lactobacilli had a significant effect on increasing learning and memory in the tested groups. It is worth mentioning that the STL results were the only parameter that showed a significant difference in the control and lactobacilli groups. We hypothesize that this fact can explain the observation that the STL test evaluates short-term memory, and the alterations in this feature manifest easily and don’t require more structural changes. Also, in the results of hippocampal histology, the absence of Aβ plaques was observed in the preventive group and the reduction of Aβ plaques in the treatment group. The findings show that our formulation of lactobacilli can reduce neurological disorders in preventive and treatment groups.

Therefore, as the pieces of evidence have demonstrated, several mechanisms, including antioxidant, anti-inflammatory, and alteration of neurotransmitter levels, contributed to the protective effects of various probiotic strains or probiotic compounds against learning and memory deficits as well as the histopathological alteration observed in various models of AD^70,71^. Nonetheless, it may be concluded that one or more of the proposed mechanisms may be responsible for our newly developed protective and therapeutic effects of lactobacilli against learning and memory deficits caused by *P. gingivalis* bacteria and histopathological improvements.

The disturbance of the homeostatic balance of cortex and hippocampus TEs levels has previously been proposed as a mechanism involved in AD, learning, and memory disturbance. Therefore, the TEs concentrations in the cortex and hippocampus tissues were assessed in the present study, and a noticeable change in TEs in the cortex and hippocampal tissue following *P. gingivalis*-induced gingival inflammation was found. More importantly, the newly developed lactobacilli rescued and improved the imbalanced TEs levels in preventive and curative administration models. The research showed that *P. gingivalis*-induced gingival inflammation altered the TEs in the brain and hippocampal tissue.

Most importantly, the TEs imbalanced in preventive and curative administration models were improved by the newly developed lactobacilli agent. These findings suggested a novel mechanism that may have contributed to the neuroprotective benefits of lactobacilli against *P. gingivalis* effect of AD-like pathology. It is important to note that while Fe levels increased, there were no differences between the lactobacilli and control groups in all other TE levels. It showed the possible accelerating role of lactobacilli mixture consumption in Fe adsorption. This finding confirmed the previous study that adding *L. plantarum* 299v (Lp299v) to fruit drinks could improve Fe adsorption^72^. Das et al. have also reported that gut microbial-derived metabolites play a crucial role in systemic iron homeostasis^73^.

From a neuroprotective and histological standpoint, the present study established a robust and advantageous lactobacilli therapeutic strategy in the preventive and curative models of *P. gingivalis*-induced gingival inflammation. Finally, given that the preventive model of lactobacilli administration performed better than the curative model, it is worthwhile to remember the adage “preventive is better than curative.”

## Conclusion

In summary, this study provides evidence on the role of lactobacilli in alleviating the gingival and cognitive abnormalities induced by *P. gingivalis* infection. Additionally, our investigation is the first to document the disruptive effects of *P. gingivalis* infection on the TEs homeostasis in the cortex and hippocampus, laying a good foundation for further assessments of the feasibility of this alteration in AD-like pathological development by *P. gingivalis*. In addition, we demonstrated both preventive and curative models of lactobacilli therapy rescued and ameliorated the brain TEs imbalance, although the preventive model showed a better performance. Finally, this study introduced a mixture of lactobacilli which may be a good candidate for prevention and curation of *P. gingivalis* infection and its abnormalities. It is worth mentioning that it could be considered an effective non-surgical therapy that doesn’t induce any pain in the patients. Further studies could be conducted to detect the mechanisms may be responsible for protective effects of our formulated lactobacilli against learning and memory deficits caused by *P. gingivalis* that may provide new insight for lactobacilli as an ideal therapeutic target.

## Data Availability

The datasets used and/or analysed during the current study available from the corresponding author on reasonable request.
